# Vaccination Coverage for Infants: Cross-Sectional Studies in Two Regions of Belgium

**DOI:** 10.1155/2014/838907

**Published:** 2014-05-26

**Authors:** Emmanuelle Robert, Michèle Dramaix, Béatrice Swennen

**Affiliations:** ^1^Research Center of Health Policy and Systems-International Health, School of Public Health, Université Libre de Bruxelles, Route de Lennik 808, 1070 Brussels, Belgium; ^2^Research Center of Epidemiology, Biostatistics and Clinical Research, School of Public Health, Université Libre de Bruxelles, Route de Lennik 808, 1070 Brussels, Belgium

## Abstract

*Methods and Objectives.* To estimate infant vaccination coverage in the French-speaking region of Belgium (Wallonia) and in the Brussels-Capital Region, two cross-sectional studies were performed in 2012. A face-to-face questionnaire was administered by trained investigators. The objective was to evaluate infant vaccination coverage retrospectively in 18- to 24-month-old children. These studies offered the opportunity to assess some factors influencing vaccine uptake in infants. *Results and Discussion.* Approximately 99% of the children had received the first dose of IPV-DTaP, 90% the fourth dose, 94% the MMR vaccine, 97% the first dose of pneumococcal vaccine, and 90% the third dose. In both regions, when fitting a logistic model, the most associated factor was attendance at maternal and child clinics (MCH). No association was observed between vaccination coverage and the mother's level of education. For the last immunization session, where the mother was a Belgian native and when she worked more hours, child was better immunized, but only in Brussels. *Conclusion.* Coverage for the fourth dose of hexavalent vaccine (DTaP-IPV-HBV/Hib) needs to be increased. Indeed, additional effort is needed to increase HIB and pertussis coverage rates because the herd immunity threshold for these two diseases has not been reached.

## 1. Introduction

Immunization coverage is a major indicator for vaccination programs. Without a vaccination registry, regular assessment should be done to measure changes in coverage and to provide avenues for action.

In Belgium, there are 3 regions, and the immunization program is organized on a regional basis but the vaccination schedule is identical for all the country and recommended by the National Health Council (NCH). All vaccines (except rotavirus vaccine) that are recommended by the NHC are provided free of charge by each regional government. Polio vaccination is the only mandatory infant vaccination. Documented polio vaccination must be returned to the municipal administration at 18 months of age. If not done, parents may incur a penalty. Since 2007, national recommendations for infant immunization include the administration of DTaP-IPV-HBV/Hib vaccine (hexavalent) at 8, 12, and 16 weeks and at 15 months, pneumococcal vaccine (PCV7) at 2, 4, and 12 months, the first dose of measles-mumps-rubella (MMR) at 12 months, and meningococcal C vaccine (MenC) at 15 months. The complete schedule includes five sessions during which it is recommended to perform several injections (e.g., session 5: Hexa4 + MenC). Parents can choose to have their child immunized in a maternal and child health clinic (MCH), which is a public organization, or by a private practitioner. Administration of the infant vaccine is free of charge when performed at the MCH. For parents who object to the DTaP-IPV-HBV/Hib vaccine, it is possible to obtain the DTaP-IPV (tetravalent) vaccine, IPV Imovax vaccine, or Hib vaccine. A fee must be paid if the vaccine is administered at a general practitioner or pediatrician's practice. At birth, a child health booklet is given to the parents. Each vaccination is recorded in it by the vaccinators. There is not yet a centralized immunization registry for 2 of the 3 regions of Belgium, namely, Brussels and Wallonia.

This paper presents the current vaccine coverage in infants aged 18–24 months and the predictors for the last doses of vaccination and for complete schedules in two of the three Belgian Regions covering 45% of the population.

## 2. Material and Methods 

### 2.1. Population and Sampling

An expanded program on immunization (EPI) two-stage cluster sampling study was conducted in Wallonia. To calculate the sample size, the margin of error for the 95% confidence interval was desired at 2.5%. Coverage rate of 91.2% (MenC) and a design effect of 1 were based on the results of the 2009 study. With these parameters the target number was 495 children. We added the nonrespondents rate of the previous study. In the first stage, 55 clusters were selected with probability proportional to size, which allowed larger municipalities to be drawn more than once. In the second stage, 12 children in each cluster were randomly selected from the municipality list. A minimum of 9 children per cluster must be reached.

The number of children selected at random in each of the 19 municipalities of Brussels-capital Region was proportional to the size of the municipality. To calculate the sample size, the margin of error for the 95% confidence interval was desired at 2.5%. Coverage rate of 91.1% (MenC) was based on the results of the 2006 study. With these parameters the target number was 499 children. We added the nonrespondents rate of the previous study.

A list of children born between 31 May and 30 November 2010 was obtained from each municipality. All of the children were aged between 18 and 24 months at the time of the surveys. Each family selected received a brief information letter announcing that an interviewer would visit them. Families were substituted with a replacement if they could not be contacted after three attempted visits. If parents were reached but refused to participate, the child was not replaced to limit the risk of selection bias, as refusal could be linked to a negative attitude against vaccination. The two studies were conducted between May and July 2012. The two regions cover 45% of the Belgian population. The databases were registered by the Commission for the Protection of Privacy in Belgium.

### 2.2. Variables and Definitions

Vaccination dates for hexavalent, pneumococcal, MMR, and meningococcal C doses since birth were transcribed from vaccination document at home. Vaccine doses that are not recorded were considered as not given. Children without vaccine record because of refusal of immunization were included in the sample as nonvaccinated. In addition to the dates of vaccination, sociodemographic determinants were asked about their age, working time, educational level, nationality of origin of the mother, gender and parity of the child, attendance at maternal and child clinics (MCH), and the use of day-care centers. To be comparable to the literature, vaccination coverage was first presented by disease. To do this, the hexavalent was broken down by disease (diphtheria, tetanus, pertussis, polio, hepatitis B, and Hib). We also added coverage with isolated vaccines (DTP, IPV, HBV, or Hib).

In contrast, for the analysis of the risk factors, to avoid redundancy, we presented complete coverage with hexavalent vaccine (Hexa4).

Vaccination coverage by vaccine dose was defined as the percentage of children for whom a vaccination date was registered on their vaccination document for that dose at the time of the study. A complete schedule was considered as 4 doses of hexavalent, 3 doses of pneumococcal, 1 dose of MMR, and 1 dose of MenC. A partial schedule was defined as a lack of at least one dose of these vaccines.

For the logistic regression, all socioeconomic variables were dichotomized. For the mother's working time, the first category included mothers who worked full time, those who were self-employed and on maternity leave. The second category included unemployed mothers or mothers without a replacement income and working part time.

### 2.3. Statistical Analysis

Coverage estimates for the two regions are presented with a 95% confidence interval. Multiple logistic regressions models were used to study each complete recommended vaccine. Variables significantly associated at *P* < 0.10, with at least one of the vaccine coverage in bivariate analysis, were included in all logistic models. The adjusted odds ratios from regression analysis are presented with 95% confidence intervals. An association was considered significant if the *P* value did not exceed 5%. Potential interactions were tested and were nonsignificant. Epi-Info 6.04d Fr (Centre for Disease Control and Prevention) was used for encoding and analyses were performed with IBM SPSS 22.0.

## 3. Results

### 3.1. Study Population

Of the 660 children originally included in the Wallonia sample, 82 (12.4%) families could not be contacted (despite the substitution). Of the 578 (87.2%) families successfully contacted (8.3% substituted once), 51 (8.8%) refused to cooperate mainly because of lack of time or interest. The vaccination documents of 519 children (78.6%) were consulted and included in the analysis. The design effect did not exceed 1.06 for any of the vaccine doses.

Of the 597 children originally included in the Brussels sample, 27 (4.5%) families could not be contacted (despite the substitution). Of the 570 (95.5%) families successfully contacted (21.7% substituted, sometimes twice), 24 (4.2%) refused to cooperate. The vaccination documents of 538 children (90.1%) were consulted and included in the analysis. The sociodemographic characteristics of the children and parents in the two samples are presented in Table 1.

The samples were representative of the Wallonia and of the Brussels-Capital Region. Indeed, the sociodemographic data were comparable to the two databases of the Perinatal Epidemiology Centre (CEPIP) which records and analyzes all statistical reports on births in the two regions [1, 2].

The Brussels-Capital Region included a greater number of foreign native mothers (76.4%), mothers with no income (24.3%), and children attending an MCH (63.4%).

### 3.2. Vaccination Coverage

One child in Wallonia and two children in Brussels had not been immunized against any disease, including the mandatory polio vaccination. The coverage rates are presented in Table 2. For each disease, the rates were constant for the first three doses. In the two regions, the last dose was systematically less administered. For IPV-DTaP, the vaccination coverage dropped from over 99% for the first dose to 90% for the fourth dose. For pneumococcal vaccine, the coverage dropped from 97% to 89% for the last dose. Any complete vaccinations reached at least 89%, as is the case for MenC, except for MMR which reached 94%. In Brussels, 3.9% of the children had not received the recommended schedule with hexavalent vaccine but other combinations (DtaP-IPV) or IPV only excluding often HBV vaccine or Hib vaccine. In Wallonia they were 2.1%.

### 3.3. Characteristics Associated with Complete Childhood Vaccination

The regression models for each complete vaccine for Wallonia are shown in Table 3. Being a first child and attendance at an MCH consultation were significantly and positively associated for all complete vaccines and complete schedules. In Wallonia, the native nationality, age, educational level, and employed situation of the mother were never associated with better vaccination coverage. Children attending a day-care center were never associated with better coverage either.

The regression models for each complete vaccine in the Brussels-Capital Region are shown in Table 4. As in Wallonia, attending an MCH consultation was significantly associated for all complete vaccines. Unlike Wallonia, being a first child was not significantly associated with any vaccine. Children of older mothers and those whose mothers worked more hours were better vaccinated with Hexa4 and MenC. Children who attended a day-care center and children of native foreign mothers were associated with better PCV7 coverage. Children who often received a complete schedule were those whose mothers worked the most.

## 4. Discussion

### 4.1. Vaccination Coverage and Herd Immunity Threshold

The vaccination coverage measured in these two studies showed rates based on reliable documented vaccination. In Wallonia, the study showed a slight increase for complete pneumococcal vaccine (+8.5%) since the last study performed in 2009 [3]. Coverage for the other vaccines remained stable.

In Brussels, the survey showed a slight increase for the MMR vaccine (+3%) and a significant increase for the pneumococcal vaccine (+80.8%) since the last study performed in 2006 [4]. At that time, the pneumococcal vaccine was not free of charge. Coverage for all the other vaccines has remained stable since 2006 in the Belgian capital.

In the two regions, the critical vaccine coverage for polio, diphtheria, mumps, and rubella has been reached. Pertussis requires coverage levels of around 92–95% and* Haemophilus influenzae* type b around 95% [5]. Therefore additional effort is needed to increase the coverage levels of Hib and pertussis. Even if some valences reached the critical values, the fourth dose of hexavalent vaccine must be increased. The WHO's very high goal (95%) in the current plan to eliminate measles and rubella [6] was almost reached in the two regions. In Flanders, the MMR coverage was 96.6% in 2012 [7] and thus the WHO's goal has been reached for some years.

Our type of sampling did not highlight the existence of groups with low measles vaccine coverage. In Belgium, anthroposophical schools, in which most of the children are unvaccinated, are mainly located in Flanders (the third region of Belgium), with just a few in Brussels and Wallonia [8, 9]. The resurgence of measles in 2011 in Belgium showed the need to maintain high vaccination coverage because other groups were affected by the measles virus in Brussels and Wallonia, for example, a Roma community and families opposed to vaccination due to fear of side effects [8].

### 4.2. Major Impact of MCH Consultations

In 2012, in Wallonia and in Brussels, attending an MCH consultation remained associated in all logistic models. The children who attended an MCH consultation were often more vaccinated. In 2012, in Flanders, the influence of this predictor was the same [10]. In the three regions of the country, we can say that attending an MCH consultation is the predictor most often and most strongly associated with any complete vaccination. MCH consultations are free. In the French-speaking community of Belgium, the children who attended MCH consultation most often came from disadvantaged backgrounds. More educated parents attended private physicians. In Flanders too, the choice of a certain administrator was associated with sociodemographic variables. A possible explanation is that an appointment with the GP or pediatrician can be organized more readily after working hours [11]. Parents who are more educated expressed concern regarding the safety of vaccines and indicated that medical doctors had little influence over the vaccination decisions they made for their children [12]. We suppose that the parents opposed to one or other vaccine attended more private physicians than MCH consultations.

### 4.3. Less Impact of Sociodemographic Determinants

In Wallonia, in 1999, no association between vaccination and most of the sociodemographic factors was demonstrated [13]. At the same time, in the Flemish community, infant vaccination coverage could not be associated with any sociodemographic factors except for hepatitis B vaccination [13]. In 2006, the only vaccine against pneumococcus was not free of charge. Parents had to pay €66 per dose. Traditional indicators of social disparities (parent's outcome, mother's education level, and attendance at day-care) persisted in the univariate and multivariate analyses for this paid-for vaccine in 2006 but disappeared for all vaccines supported by the community [14]. Only for this paid-for vaccine were children attending private physicians most frequently vaccinated (52.1% for private physicians versus 23.3% MCH).

In Wallonia, in 2012, it would seem that vaccination policy and free access to vaccine largely reduce health inequalities. Indeed, children with a less advantaged socioeconomic background (unemployed mother or working part time, mother's lower level of education, and foreign native mothers) were not less vaccinated than others. However, in Brussels, differences existed for some factors. When the mother was older or when she worked more, the child was more likely to be vaccinated with Hexa4 and MenC (administered during the same session). The mother's employment situation affected the complete schedule too. In Flanders, in 2012, this predictor was significant for having MMR and PCV7 [10]. However, the mother's education level did not influence the vaccination coverage. While, in Wallonia, the mother's native nationality was never associated with any complete vaccination; in Brussels this association only existed for the final PCV7 dose [15].

In the Netherlands, the vaccination policy is reported to be associated with reduced health inequalities [16]. In the US, mothers younger than 30 years old are associated with not receiving all vaccines [17]. Other studies show that better coverage correlates with the most advantaged backgrounds [18]. The number of vaccines recommended and whether they are provided free of charge by the government, the provision of MCH consultations, and the reimbursement system for private medical fees are so different from one country or region to another that it is not easy to compare the impact of socioeconomic predictors on the quality of coverage.

In Wallonia, the first child was systemically associated with a higher vaccination rate. This predictor is already known in the literature [18] but, in Brussels, it does not appear to be associated with a higher vaccination rate. In Flanders, the first born child is generally better vaccinated than the following children [10, 19].

### 4.4. Refusal and Particular Schedules

Surveys of immunization coverage in Belgium did not reveal a significant percentage of parents who oppose vaccination. The rates in the different surveys do not seem to exceed 1% for the only mandatory vaccine in the country. Outright refusal of vaccination was an exception in Flanders and in Wallonia and Brussels too. Only one child in Wallonia and two in Brussels were found. Other countries, like the Netherlands, also have very low or extremely low refusal rates [16]. Given the rarity of these cases it is impossible to profile these children in Belgium. Only large studies can provide the profile of these children. The profile may indeed be different for children who are partially vaccinated [6, 12]. A higher proportion of mothers of unimmunized infants were educated to degree level or above, were older, and had a better household income [6, 12].

In Brussels, 3.9% of the children had not received the recommended schedule but an alternative schedule (DTPa-IPV, IPV). In Wallonia, 2.1% of the children had received an alternative schedule. In these two regions, the use of separate vaccines is more frequent when the mother is Belgian and when the parents are more educated. These particular schedules were administered by private physicians. It should be noted that the father's level of education is more strongly associated with these particular patterns than the mother's.

### 4.5. Study Limits

The samples sizes of the two surveys reached the desired size and were representative of the two regions. It remains unclear as to whether or not the children whose parents refused the survey were undervaccinated. Indeed, we had no idea of the profile of parents refusing to participate to the study. We do not know if the refusal is in link with a negative attitude about vaccination. However, we believe that this bias is limited because the parents did not know the topic of the survey before they accept or refuse the interview. They were informed of a survey about infancy. Children illegally resident in the two regions were not included in the surveys. Those children may be less vaccinated than resident; however, according to the program policy they have free access to the MCH services.

## 5. Conclusions

Since 1989, regular cross-sectional studies with the same methodology have been conducted in Wallonia (nine surveys) and the Brussels-Capital Region (four surveys since 1995), covering 45% of Belgium's population. These studies have made it possible to not only measure the vaccine coverage, but also to study the sociodemographic variables associated with the vaccination status of the children.

In the absence of a centralized immunization registry, these studies are the only population-based measures that give the program a major indicator to follow and evaluate its efforts to cover the vaccination of preventable diseases.

Access to immunization services is an important factor in explaining health inequalities. In the two regions, free access to MCH service and to free vaccines recommended by the Heath Council are two major factors that have dramatically reduced immunization inequalities. However, the significant (around 9%) decrease in vaccination coverage after twelve months of age must be a concern for the program. To alleviate the burden of vaccine preventable diseases the critical vaccination threshold specific to each disease needs to be achieved. In 2012, Wallonia and the Brussels-Capital Region with coverage above 94% almost reached the WHO objective of 95% coverage for the first dose of MMR for the first time.

## Figures and Tables

**Table 1:** Main characteristics of the two sampled (Wallonia and Brussels) children and their mothers (*n*, %).

Characteristics	Wallonia		Brussels	
	*n*	%	*n*	%
Mother's age				
16–30	249	49.1	191	35.9
>30	258	50.9	341	64.1
Mother's educational level				
Maximum secondary level	293	57.8	296	56.1
Higher than secondary level	214	42.2	233	43.9
Parity				
1	221	42.8	215	40.2
2	171	33.1	169	31.6
3	82	15.9	98	18.3
>3	42	8.2	53	9.9
MCH consultation				
Yes	278	53.8	340	63.4
No	239	46.2	196	36.6
Attendance at day-care				
Yes	293	56.7	231	43.1
No	224	43.3	305	56.9
Mother's birth nationality				
Belgian	372	72.1	127	23.6
Other	144	27.9	410	76.4
Mother's employment situation				
Full time salaried	135	27.2	118	22.3
Part-time salaried	130	26.2	86	16.2
Self-employed	28	5.6	20	3.8
Unemployed	133	26.8	159	30.0
Maternal leave	19	3.8	18	3.4
Without a replacement income	52	10.5	129	24.3
Gender				
Male	267	51.6	278	51.7
Female	250	48.4	260	48.3

**Table 2:** Percentage vaccination coverage (%) and 95% confidence interval of all recommended vaccinations in children aged 18–24 months, Wallonia and Brussels.

	IPV-DTaP	Hib	HBV	MMR	MenC	Pneumococcal
Wallonia	(519)					
Dose1	99.8 (99.4–100)	99.2 (98.5–100)	98.1 (96.9–99.3)	94.4 (92.4–96.4)	89.6 (87.0–92.2)	97.1 (95.7–98.6)
Dose2	99.2 (98.5–100)	98.5 (97.4–99.5)	97.2 (96.2–98.8)			97.1 (95.7–98.6)
Dose3	99.2 (98.5–100)	98.5 (97.4–99.5)	97.2 (96.2–98.8)			89.2 (86.5–91.9)
Dose4	90.4 (87.8–92.9)	89.4 (86.8–92.1)	89.2 (86.5–91.9)			

Brussels	(538)					
Dose1	99.6 (99.1–100)	97.3 (95.8–98.6)	96.7 (95.1–98.2)	94.1 (92.1–96.1)	89.4 (87.0–92.2)	97.0 (95.6–98.5)
Dose2	99.3 (98.5–100)	97.6 (96.6–98.9)	96.8 (95.4–98.3)			95.5 (93.8–97.3)
Dose3	98.7 (97.7–99.7)	96.7 (95.1–98.2)	96.3 (94.7–97.9)			90.1 (87.6–92.7)
Dose4	91.1 (88.7–93.5)	90.1 (87.6–92.7)	89.6 (87.0–92.2)			

**Table 3:** Observed coverage's (%) and adjusted odds ratio (a OR, 95% CI, *P* value) for complete vaccine and for total recommended schedule in Wallonia.

Wallonia (*n* = 481)	Hexa4			RRO			MenC			Pn3			Complete schedule		
	Coverage	a OR	*P* value	Coverage	a OR	*P* value	Coverage	a OR	*P* value	Coverage	a OR	*P* value	Coverage	a OR	*P* value
Mother's age (years)															
16–30	89.2	1		95.2	1		91.2	1		89.2	1		83.1	1	
>30	88.8	1.4 (0.7–2.7)	0.3	94.2	0.9 (0.3–2.3)	0.8	88.4	0.9 (0.5–1.7)	0.7	89.1	1.1 (0.6–2.1)	0.8	80.6	0.9 (0.5–1.5)	0.9
Mother's educational level															
Maximum secondary level	86.7	1		92.5	1		88.3	1		85.3	1		80.8	1	
Higher than Secondary level	90.2	1.5 (0.7–3.1)	0.3	95.1	2.7 (0.9–8.4)	0.08	90.2	1.8 (0.8–4.0)	0.1	90.4	1.9 (0.9–4.0)	1.0	82.9	1.2 (0.7–2.3)	0.5
Parity															
1	**94.1**	**2.8** **(1.3**–**5.7)**	**0.006**	**95.9**	**5.6** **(1.5**–**21.2)**	**0.01**	**93.2**	**2.3** **(1.1**–**4.9)**	**0.03**	**93.2**	**2.8** **(1.3**–**6.0)**	**0.007**	**86.4**	**2.0** **(1.1**–**3.6)**	**0.02**
>1	**85.6**	**1**		**93.3**	**1**		**86.9**	**1**		**86.2**	**1**		**78.5**	**1**	
MCH consultations															
Yes	**95.0**	**5.1** **(2.5**–**10.2)**	**<0.001**	**98.6**	**8.1** **(2.3**–**28.8) **	**<0.001**	**94.6**	**3.7** **(1.8**–**7.3)**	**0.001**	**93.5**	**2.8** **(1.5**–**5.1) **	**<0.001**	**89.2**	**3.2** **(1.9**–**5.4)**	**<0.001**
No	**82.8**	**1**		**89.5**	**1**		**83.7**	**1**		**84.5**	**1**		**73.6**	**1**	
Mother's birth nationality															
Belgian	89.2	1.0 (0.5–2.1)	0.9	93.8	0.7 (0.3–1.8)	0.5	89.0	0.9 (0.4–1.9)	0.8	89.0	0.9 (0.4–1.9)	0.9	81.2	0.8 (0.5–1.5)	0.6
Other	89.6	1		95.8	1		91.7	1		89.6	1		84.0	1	
Mother's working time															
Unemployed, part time	88.9	1		95.6	1		90.8	1		88.6	1		81.9	1	
Full time, self-employed	89.0	0.7 (0.4–1.5)	0.4	93.4	0.6 (0.2–1.6)	0.3	87.9	0.6 (0.3–1.3)	0.2	90.7	1.1 (0.5–2.2)	0.8	82.4	1.0 (0.6–1.7)	0.9
Attendance at day-care															
Yes	90.8	1.7 (0.9–3.4)	0.1	93.2	0.7 (0.3–2.1)	0.6	89.4	1.2 (0.6–2.5)	0.5	88.7	1.2 (0.4–1.7)	0.7	82.3	1.3 (0.7–2.3)	0.4
No	87.1	1		96.0	1		89.7	1		89.7	1		81.2	1	

Not included in the models: infant's sex and father's educational level.

**Table 4:** Observed coverage's (%) and adjusted odds ratio (a OR, 95% CI, *P* value) for complete vaccine and for total recommended schedule in Brussels.

Brussels (*n* = 518)	Hexa4			RRO			MenC			Pn3			Complete schedule		
	Coverage	a OR	*P* value	Coverage	a OR	*P* value	Coverage	a OR	*P* value	Coverage	a OR	*P* value	Coverage	a OR	*P* value
Mother's age (years)															
16–30	**86.9**	**1**		94.2	1		**85.3**	**1**		92.7	1		81.2	1	
>30	**90.9**	**1.9** **(1.0–3.5)**	**0.04**	94.1	1.5 (0.6–3.6)	0.4	**91.8**	**2.4** **(1.3**–**4.5)**	**0.006**	89.1	0.8 (0.4–1.6)	0.5	85.0	1.5 (0.9–2.7)	0.1
Mother's educational level															
Maximum secondary level	88.2	1		96.1	1		88.2	1		92.1	1		81.6	1	
Higher than secondary level	90.2	1.1 (0.6–2.2)	0.7	93.4	0.5 (0.2–1.6)	0.3	90.0	1.1 (0.5–2.1)	1.0	89.7	0.9 (0.4–2.0)	0.9	84.4	1.1 (0.6–2.1)	0.7
Parity															
1	90.2	1.2 (0.6–2.2)	0.5	95.8	1.9 (0.8–4.4)	0.2	89.8	1.1 (0.6–2.1)	0.8	91.2	1.4 (0.7–2.6)	0.4	82.8	1.0 (0.6–1.6)	0.9
>1	89.2	1		92.9	1		89.2	1		89.5	1		83.9	1	
MCH consultations															
Yes	**93.2**	**3.4** **(1.8**–**6.3) **	**0.001**	**97.9**	**6.6** **(2.7**–**16.5) **	**0.001**	**93.2**	**3.4** **(1.8**–**6.3)**	**0.001**	**95.6**	**4.8** **(2.4**–**9.4)**	**0.001**	**90.6**	**4.6** **(2.7**–**7.8)**	**0.001**
No	**83.2**	**1**		**87.8**	**1**		**83.2**	**1**		**81.1**	**1**		**71.4**	**1**	
Mother's birth nationality															
Belgian	85.8	0.6 (0.3–1.3)	0.2	91.3	0.7 (0.3–1.8)	0.5	86.6	0.7 (0.3–1.5)	0.4	**84.3**	**0.5** **(10.2**–**1.0)**	**0.04**	78.7	0.7 (0.4–1.2)	0.2
Other	90.7	1		95.1	1		90.5	1		**92.2**	**1**		85.1	1	
Mother's working time															
Unemployed, part time	**88.0**	**1**		94.4	1		**88.0**	**1**		90.6	1		**81.3**	**1**	
Full time, self-employed	**92.9**	**2.4** **(1.1**–**5.1) **	**0.03**	93.6	1.3 (0.5–3.1)	0.6	**92.4**	**2.3** **(1.1**–**5.0)**	**0.04**	89.1	1.0 (0.5–2.1)	0.9	**88.5**	**2.3** **(1.2**–**4.3)**	**0.01**
Attendance at day-care															
Yes	89.2	0.9 (0.5–1.9)	0.9	93.5	1.1 (0.5–2.8)	0.8	89.2	0.9 (0.2–3.5)	0.7	**90.5**	**2.1** **(1.0**–**4.5)**	**0.05**	84.0	1.2 (0.7–2.2)	0.5
No	89.8	1		95.1	1		90.2	1		**90.2**	**1**		83.6	1	

Not included in the models: infant's sex and father's educational level.
